# Generation and Phenotype Identification of *PAX4* Gene Knockout Rabbit by CRISPR/Cas9 System

**DOI:** 10.1534/g3.118.300448

**Published:** 2018-06-27

**Authors:** Yuanyuan Xu, Yong Wang, Yuning Song, Jichao Deng, Mao Chen, Hongsheng Ouyang, Liangxue Lai, Zhanjun Li

**Affiliations:** Jilin Provincial Key Laboratory of Animal Embryo Engineering, Jilin University, Changchun 130062, China

**Keywords:** CRISPR/Cas9, gene knockout, *PAX4*, rabbit

## Abstract

Paired-homeodomain transcription factor 4 (*PAX4*) gene encodes a transcription factor which plays an important role in the generation, differentiation, development, and survival of insulin-producing β-cells during mammalian pancreas development. *PAX4* is a key diabetes mellitus (DM) susceptibility gene, which is associated with many different types of DM, including T1DM, T2DM, maturity onset diabetes of the young 9 (MODY9) and ketosis prone diabetes. In this study, a novel *PAX4* gene knockout (KO) model was generated through co-injection of clustered regularly interspaced short palindromic repeats (CRISPR)-associated protein 9 (Cas9) mRNA/sgRNA into rabbit zygotes. Typical phenotypes of growth retardation, persistent hyperglycemia, decreased number of insulin-producing β cells and increased number of glucagon-producing α cells were observed in the homozygous *PAX4* KO rabbits. Furthermore, DM associated phenotypes including diabetic nephropathy, hepatopathy, myopathy and cardiomyopathy were also observed in the homozygous *PAX4* KO rabbits but not in the wild type (WT) controls and the heterozygous *PAX4* KO rabbits. In summary, this is the first *PAX4* gene KO rabbit model generated by CRISPR/Cas9 system. This novel rabbit model may provide a new platform for function study of *PAX4* gene in rabbit and gene therapy of human DM in clinical trails.

*PAX4* (paired-homeodomain transcription factor 4) gene belongs to the Pax gene families, encodes a transcription factor which plays major roles in the generation, differentiation, development, and survival of insulin-producing β cells during mammalian pancreas development (Napolitano *et al.* 2015; Lorenzo *et al.* 2017). It has been confirmed that *PAX4* is a key diabetes mellitus (DM) susceptibility gene, for single-nucleotide polymorphisms (SNPs) and mutations in *PAX4* caused maturity onset diabetes of the young 9 (MODY9) (Anık *et al.* 2015), increased susceptibility to type 2 diabetes mellitus (T2DM) (Sujjitjoon *et al.* 2016a), and were also associated with type 1 diabetes mellitus (T1DM) (Biason-Lauber *et al.* 2005) and ketosis prone diabetes (Mauvais-Jarvis *et al.* 2004).

The importance of *PAX4* gene in the development of DM has been manifested in the *PAX4* gene knockout (KO) mice (Sosa-Pineda *et al.* 1997). Homozygous *PAX4* KO mice showed a lack of mature insulin-producing β cells and a severe diabetic syndrome after birth. However, heterozygous *PAX4* KO mice did not exhibited any obvious abnormalities, since they possessed normal amount of insulin-producing β cells and did not develop DM (Sosa-Pineda *et al.* 1997). In addition, overexpression of *PAX4* gene can stimulate β cell proliferation and increase their resistance to hyperglycemia induced apoptosis in adult islets (Hu He *et al.* 2011). Therefore, *PAX4* can be used as an important target in developing new therapies of human DM.

Up to now, several *PAX4* mutations associated with DM have been reported, including six missense mutations (R121W, R129W, R133W, R164W, R192H, and P321H) (Shimajiri *et al.* 2001; Mauvais-Jarvis *et al.* 2004; Plengvidhya *et al.* 2007; Hu He *et al.* 2011; Sujjitjoon *et al.* 2016a), a 39-bp deletion in exon 3 (Jo *et al.* 2011), and an splice acceptor site mutation of IVS7-1G > A (Sujjitjoon *et al.* 2016b). These mutations disrupt the normal function of *PAX4* gene, resulting in dysfunction of pancreatic β cells and subsequent insulin secretion, and finally lead to the development of DM (Brun and Gauthier 2008; van der Meulen and Huising 2015).

Nowadays, though mouse models have been widely used to simulate human diseases for clarifying important pathological mechanisms and identifying possible therapeutic targets, they cannot fully recapitulate human characteristics due to differences in physiological traits and gene expression (Li and Li 2012). Alternatively, because of the intermediate size and similar disease characteristics with humans, the rabbits are extensively used as a suitable human disease model, especially in metabolic disease research (Bosze *et al.* 2003; Wang *et al.* 2014). Therefore, despite a *PAX4* gene KO mouse model has been reported and aided in understanding the pancreatic development, β cell development and function (Sosa-Pineda *et al.* 1997), there is still a pressing need to generate novel animal models which are larger and have closer phylogenetic relationships with humans to bypass these limitations shown in mouse models.

In this study, a novel *PAX4* gene KO rabbit model was generated by cytoplasmic microinjection of CRISPR/Cas9 mRNA into zygotes. Typical phenotypes of growth retardation, persistent hyperglycemia, decreased number of insulin-producing β cells, increased number of glucagon-producing α cells, and diabetic complications including diabetic nephropathy, hepatopathy, myopathy and cardiomyopathy were identified in this rabbit model. This novel rabbit model may provide a new platform for function study of *PAX4* gene in rabbit and gene therapy of human DM in clinical trails.

## Materials and methods

### Ethical statement

New Zealand rabbits were obtained from the Laboratory Animal Center of Jilin University (Changchun, China). All rabbit experiment protocols were conducted under the approval of the Animal Care Center and Use Committee of Jilin University.

### sgRNA design, vector construction and *in vitro* transcription

The protocols for sgRNA design, vector construction and *in vitro* transcription have been described in detail in our previous published protocols (Sui *et al.* 2016). First, the sgRNAs targeting rabbit *PAX4* gene (NC_013675.1) were designed using the online CRISPR Design Tool (http://crispr.mit.edu/) as previously described (Cong *et al.* 2013). Then, the complementary oligo sgRNAs were cloned into the *BbsI* sites of a Puc57-T7-sgRNA cloning vector (Addgene ID 51306). The amplified PCR products of Puc57-T7-sgRNA vector were *in vitro* transcribed using the MAXIscript T7 Kit (Ambion, USA) and purified with the miRNeasy Mini Kit (Qiagen, Germany) according to the manufacturers’ instruction. Meanwhile, the 3xFLAG-NLS-SpCas9-NLS vector (Addgene ID 48137) was linearized with *NotI* and *in vitro* transcribed with the mMessage mMachine SP6 Kit (Ambion) and the RNeasy Mini Kit (Qiagen).

### Embryo collection, microinjection and embryo transfer

Microinjection of pronuclear-stage embryos and embryo transfer were carried out based on the protocols described in our published papers (Yuan *et al.* 2016). In brief, 6-8 month female New Zealand rabbits were intravenously injected with FSH (50 IU) for superovulation every 12 h for 3 days. After the last injection, the female rabbits were naturally mated with the male rabbits and then received an injection of 100 IU of human chorinonic gonadotrophin (hCG). 18h after the hCG injection, the female rabbits were killed, and the oviducts were washed with 5 ml DPBS-BSA for zygote collection. Rabbit zygotes at the pronuclear stage were collected and transferred into the oocyte manipulation medium. Meanwhile, a mixture of *in vitro* transcribed Cas9 mRNA (200 ng/μl) and sgRNA (40 ng/μl or 20 ng/μl) was co-injected into the cytoplasm of the zygotes. Then the injected embryos were transferred and cultured in EBSS medium at 38.5°, 5% CO_2_ and 100% humidity conditions for 30-60 min. Finally, approximately 30-50 injected zygotes were transferred into the oviducts of surrogate rabbits.

### Mutation detection in pups by PCR and Sanger sequencing

TIANamp Genomic DNA Kit (Tiangen, China) was used to isolate the genomic DNA of the newborn pups according to the manufacturers’ instruction. PCR primers used to detect the *PAX4* gene mutations were as follows: F, 5′ - GTAGTCTTCTGTCCATGCCTTAC -3′, and R, 5′- CCACCTGCTACAACCCTAAAT -3′.

PCR products were then gel-purified and cloned into the pGM-T vector (Tiangen), at least 10 positive plasmid clones were sequenced and the sequences were analyzed using DNAman software.

### Off-target analysis

To test whether off-target mutations occurred in *PAX4* KO rabbits, potential off-target sites (POTS) of the sgRNAs were predicted with the online CRISPR Design Tool (http://crispr.mit.edu/), and the top 5 POTS for each sgRNA were selected. Then the PCR products of these POTS were subjected to T7E1 assay and Sanger sequence analysis. Primers are listed in Table S1.

### T7 endonuclease I (T7E1) assay

The T7E1 assay was performed as previously described (Guschin *et al.* 2010). Briefly, PCR products mentioned above were purified with TIANgel Midi Purification Kit (Tiangen) and then denatured and annealed in NEBuffer 2 (NEB, USA) using a thermocycler. Hybridized PCR products were digested with T7 endonuclease I (NEB) for 30 min at 37° and then analyzed with 2% agarose gel electrophoresis.

### Quantitative real-time PCR (qPCR)

Total RNA was extracted from the pancreas of 3-day-old WT and *PAX4* KO rabbits using TRNzol-A+ reagent (Tiangen), then treated with DNase I (Fermentas) and reverse-transcribed into cDNA using the BioRT cDNA First-Strand Synthesis Kit (Bioer Technology, China). qPCR was performed using the ABI PRISM 7500 (Applied Biosystems, USA), and relative gene expression was determined using the ∆∆CT method, which was normalized to that of the GAPDH gene. Primers for qPCR are listed in Table S2.

### Blood glucose, body weight, urinalysis and statistics

The blood glucose and body weight of the WT rabbits and the *PAX4* KO rabbits were measured everyday until the homozygous *PAX4* KO rabbits died. The blood samples were collected from the ear vein of the rabbits and the blood glucose was measured with the Roche blood glucose monitor (Glucotrend 2). Urinalysis was performed with the urine eight couplet test paper (Gaoerbao, China). All data were expressed as mean± SEM, at least three individuals of each genotype were used in all experiments. The data were analyzed with the Student’s *t*-test using Graphpad Prism software 7.0. *P* < 0.05 was considered statistically significant.

### Histology analysis and immunohistochemistry (IHC)

Pancreas, kidney, liver, lung, spleen, retina, skeletal muscle and cardiac muscle tissues of the WT rabbits and the *PAX4* KO rabbits were fixed with 4% paraformaldehyde, embedded in paraffin wax and then cut into 4 μm sections. For histology analysis, the sections were stained with hematoxylin and eosin (H&E). For IHC, immunohistochemical analysis of the pancreas sections were performed as previously described (Beucher *et al.* 2012). The following antibodies and reagents were used in this study: primary anti-insulin antibody (1:300, Bioss, China), primary anti-glucagon antibody (1:300, Boster, China), UltraSensitiveTM SP (Mouse/Rabbit) IHC Kit (MXB, China) and DAB Kit. Then the stained sections were analyzed and imaged with microscope (Nikon ts100).

### Data availability

The authors state that all data necessary for confirming the conclusions obtained in this article are fully represented within the article. Supplemental material available at Figshare: https://doi.org/10.25387/g3.6279584.

## Results

### Generation of *PAX4* KO rabbits using CRISPR/Cas9 system

**Figure 1:**
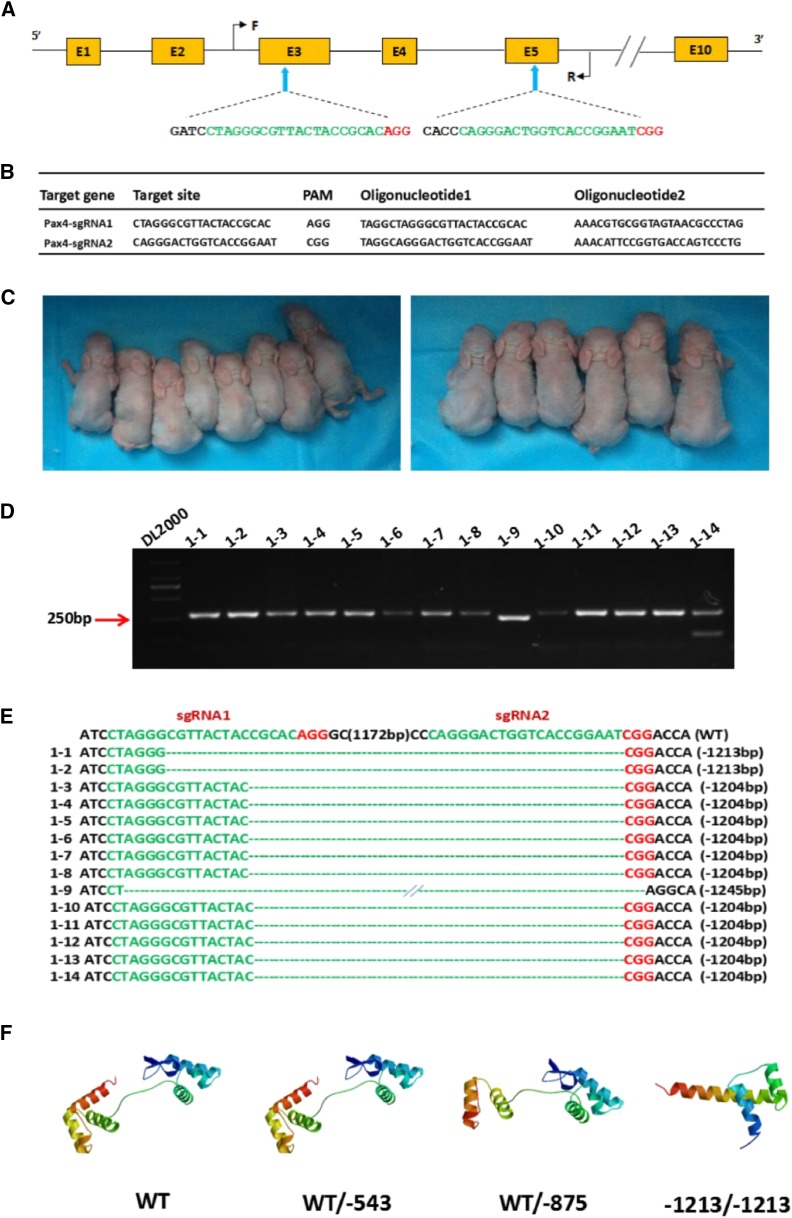
Generation of the *PAX4* knockout rabbits using the CRISPR/Cas9 system. (A) Schematic diagram of the 2 sgRNA target sites located in the exon 3 and 5 of the rabbit *PAX4* locus. *PAX4* exons are indicated by yellow rectangles; target sites of the two sgRNA sequences, sgRNA1 and sgRNA2, are highlighted in green; protospacer-adjacent motif (PAM) sequence is highlighted in red. Primers F and R were used for mutation detection in pups. (B) Target sequences of the two sgRNAs and complementary oligo sgRNAs. (C) Photographs of the *PAX4* KO rabbits generated by CRISPR/Cas9 system. (D) Mutation detection by PCR in pups 1–14. (E) Mutation detection by T-cloning and Sanger sequencing in pups 1-14 with modified *PAX4* gene. The wild type sequence is shown at the top of the targeting sequence. The sgRNA sequences are shown in green; PAM sites are highlighted in red. WT: wild type control; deletions “-”. (F) Computer modeling of PAX4 3D structure and impact of the *PAX4* mono-allelic and bi-allelic mutants at the target loci. WT: structure of non-mutant *PAX4* gene; WT/-543 and WT/-875: *PAX4* gene with mono-allelic mutation; -1213/-1213: *PAX4* gene with bi-allelic mutation.

According to the evolutionary relationship provided by NJ tree of *PAX4* gene, we found that *PAX4* gene is conservative in eutherian lineages and the rabbit *PAX4* gene has close affinity with that of rodents and primates (Figure. S1). Besides, due to the advantages on construction of gene knockout rabbits in our laboratory, we chose rabbit *PAX4* gene as the target of gene knockout. To disrupt the function of *PAX4* gene in rabbits, two sgRNAs targeting the third and the fifth exon of *PAX4* gene were designed. The target sites are shown in Figure. 1A and 1B.

Then, to generate *PAX4* KO rabbits, the *in vitro* transcribed Cas9 mRNA and sgRNA were co-injected into rabbit zygotes, and a total of 210 injected zygotes (pronuclear stage) were transferred into the oviducts of 4 surrogate rabbits, respectively (Table 1). After 30 days gestation, four recipients gave birth to 22 live pups. The genomic DNA from each pups was isolated, and mutations were detected by PCR and Sanger sequencing. As shown in Table 1, Figure 1D-1E and Figure S3, 15 newborn pups carried a biallelic *PAX4* mutation. Meanwhile, the predicted 3D models showed that the PAX4 protein structure was completely destroyed in the *PAX4* KO rabbits with 1213 bp indels, compared with that of the WT rabbits and the *PAX4* KO rabbits with 543 bp indels and 875 bp indels (Figure 1F).

**Table 1:** Summary of the *PAX4* KO rabbits generated by CRISPR/Cas9

Recipients	gRNA/Cas9 mRNA (ng/μl)	Embryos transferred	Pregnancy	Pups obtained (% transferred)	Pups with mutations (% pups)	Bi-allelic modified (% pups)	Pups with hyperglycemia (% pups)
1	40/200	56	YES	8 (14.3%)	8 (100%)	8 (100%)	8 (100%)
2	40/200	52	YES	6 (11.5%)	6 (100%)	6 (100%)	6 (100%)
3	20/200	52	YES	7 (13.5%)	4 (57.1%)	1 (25%)	1 (25%)
4	20/200	50	YES	1 (2%)	1 (100%)	0	0
Total		210	100%	22 (10.5%)	19 (86.4%)	15 (68.2%)	15 (68.2%)

**Figure 2:**
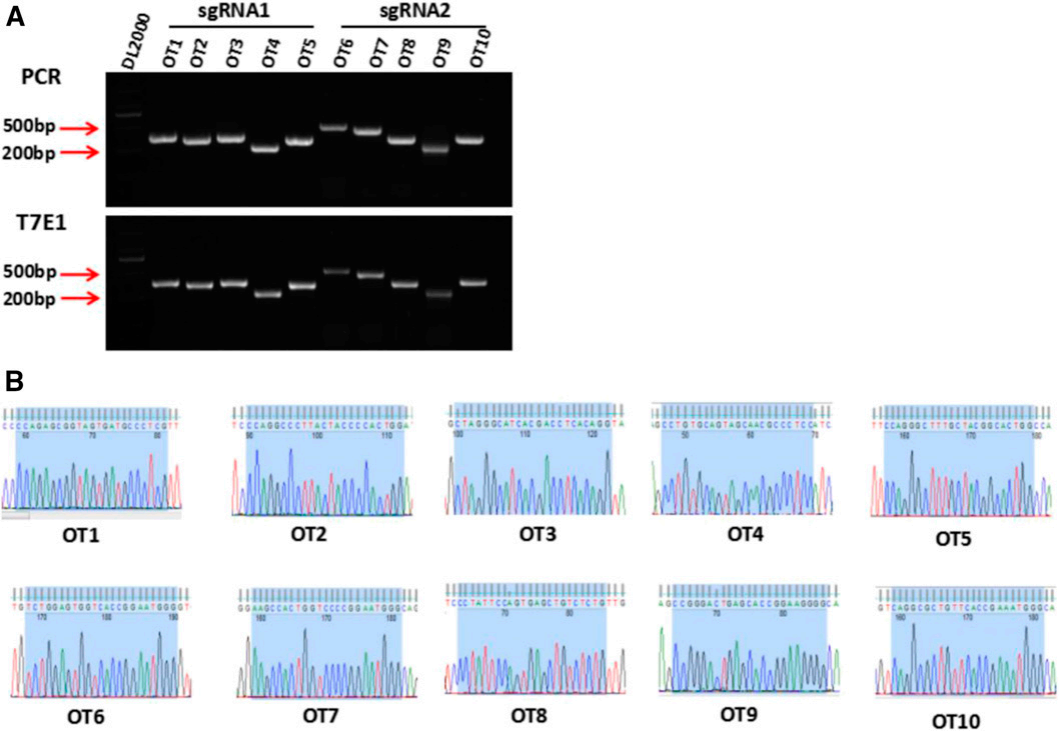
Off target detection in the *PAX4* knockout rabbits. (A) T7E1 cleavage analysis of ten potential off-target sites (POTS) for sgRNA1 and sgRNA2. 1-5 represents the five POTS for sgRNA1, 6-10 represent the five POTS for sgRNA2. (B) Chromatogram sequence analysis of five POTS for sgRNA1 and sgRNA2 using PCR products in founders. The sequences of the POTS and the PAM are represented in shadow.

Besides, off-target effect was detected in the *PAX4* KO rabbits. 5 potential off-target sites (POTs) for each sgRNA were detected by T7E1 cleavage assay and Sanger sequencing. As shown in Figure 2A and 2B, none of the sequencing reads had mutations. The information about the POTS was listed in Table S1.

These results together showed that *PAX4* KO rabbits were successfully generated by CRISPR/Cas9 system, and off-target mutations were effectively eliminated by co-injection of Cas9 mRNA and sgRNA into rabbit zygotes.

### Phenotype characterization of *PAX4* KO rabbits

After successfully generated the *PAX4* KO rabbits, we first detected the *PAX4* mRNA expression level of the KO rabbits by qPCR. As expected, compared with the WT rabbits, the *PAX4* mRNA expression level was significantly reduced in the heterozygous *PAX4* KO rabbits (PAX4^+/−^ rabbits) and homozygous *PAX4* KO rabbits (PAX4^−/−^ rabbits) (Figure. S2).

**Figure 3:**
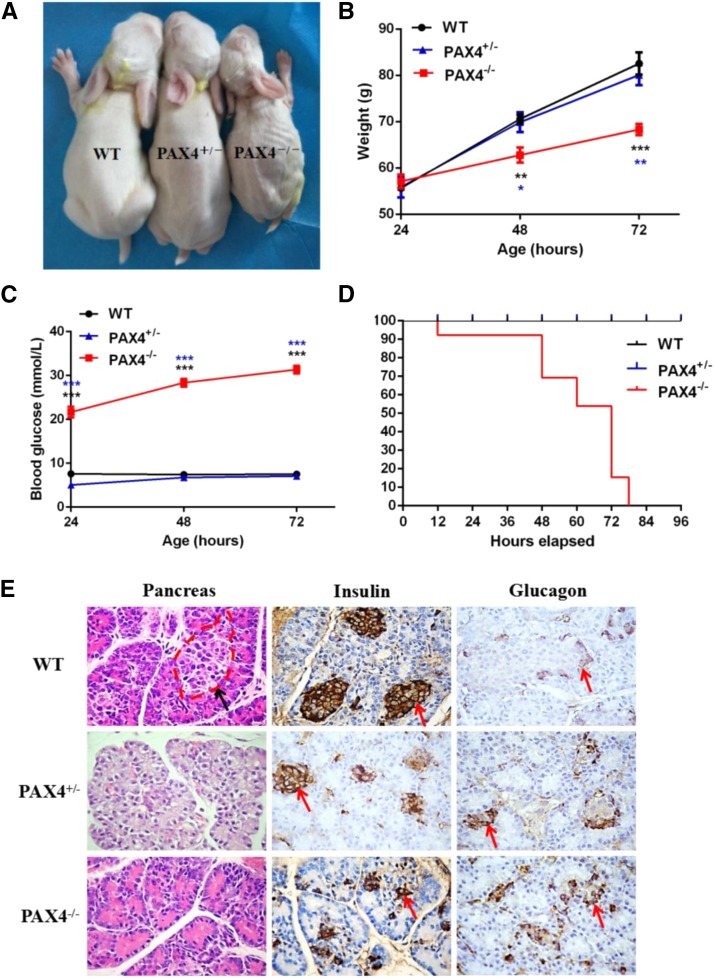
Phenotypes identification of *PAX4* KO rabbits. (A) Photographs of the WT and *PAX4* KO rabbits at the age of 3 days old. (B) Body weight of the *PAX4* KO and WT rabbits from newborn to 72 hr. (C) Blood glucose levels of the *PAX4* KO and WT rabbits from newborn to 72 hr. *P* < 0.05 was considered statistically significant, *, *P* < 0.05, **, *P* < 0.01, ***, *P* < 0.005. (D) Survival curves of the *PAX4* KO and WT rabbits. (E) H&E-staining, insulin immunohistochemistry and glucagon immunohistochemistry of the pancreas sections from the *PAX4* KO and WT rabbits. WT: wild type control; PAX4^+/−^, heterozygous *PAX4* gene knockout rabbits; PAX4^−/−^, homozygous *PAX4* gene knockout rabbits.

Then, the phenotypes caused by *PAX4* gene mutations were examined in the KO rabbits. As shown in Figure. 1C and Figure. 3B, newborn *PAX4* KO rabbits appeared normal and can’t be distinguished from the WT rabbits. However, 2 days later, the PAX4^−/−^ rabbits showed detectable growth retardation in comparison with the WT rabbits and the PAX4^+/−^ rabbits (Figure. 3A and 3B). In addition, newborn PAX4^−/−^ rabbits manifested significantly elevated blood glucose levels. And the blood glucose levels continued to elevate during the next 2 days, compared with that of the WT rabbits and the PAX4^+/−^ rabbits (Figure 3C). Meanwhile, simple urinalysis with urine analysis test papers also showed increased urin glucose levels and urin protein levels of the PAX4^−/−^ rabbits (Table S3). Furthermore, the PAX4^−/−^ rabbits died soon after birth due to severe hyperglycemia, and finally all of them died within 4 days (Figure 3D). However, the PAX4^+/−^ rabbits did not exhibit any obvious abnormalities and survived to adulthood.

H&E staining of pancreas sections showed that, compared with the normal pancreatic islet structure of WT rabbits, destroyed, incomplete pancreatic islet structure was observed in the KO rabbits (Figure 3E). Furthermore, insulin immunostaining of pancreas sections exhibited typical islet structure and distribution of insulin-producing β cells within the islets of the WT rabbits. While in the KO rabbits, only a few dispersed insulin-producing β cell mass or single β cell was observed (Figure 3E). Besides, glucagon immunostaining of pancreas sections showed that the KO rabbits contained a larger number of glucagon-producing α cells, which were abnormally clustered (Figure 3E). Significantly, all these changes were even more serious in the PAX4^−/−^ rabbits in comparison with the PAX4^+/−^ rabbits.

These results suggest that inactivation of *PAX4* gene in rabbits can induce persistent hyperglycemia caused by deficient formation of insulin-producing β cells, leading to lethality of the PAX4^−/−^ rabbits.

### Histopathological changes of *PAX4* KO rabbits

**Figure 4:**
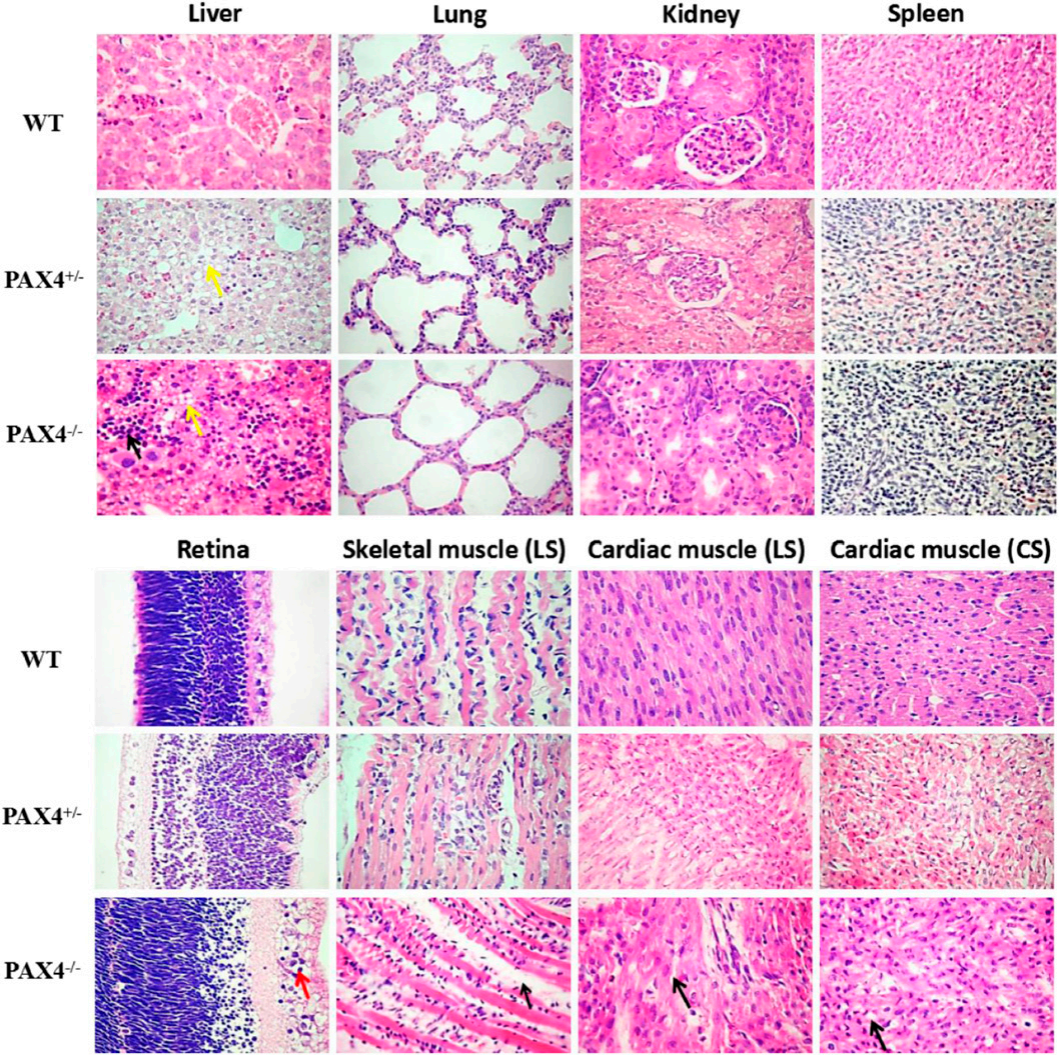
Histopathological changes of *PAX4* knockout rabbits. H&E-staining of liver, lung, kidney, spleen, retina, skeletal muscle and cardiac muscle tissues from *PAX4* KO and WT rabbits. LS: longitudinal section; CS: cross section.

As far as we know, persistent hyperglycemia resulted in development of diabetic nephropathy, hepatopathy, retinopathy, myopathy and cardiomyopathy in patients with DM (Leslie *et al.* 2016). In this study, to determine whether *PAX4* KO rabbits can develop these diabetic complications and other histopathological abnormalities, H&E staining of the liver, lung, kidney, spleen, retina, skeletal muscle and cardiac muscle was assessed. As shown in Figure 4, compared with the WT rabbits: (1) numerous inflammatory cells infiltration of the liver and hepatocyte steatosis were observed in the PAX4^−/−^ rabbits; (2) slight alveolar expansion of the lung was observed in the PAX4^−/−^ rabbits; (3) decreased numbers of glomerular, vacuolation of renal tubular epithelial cells, and smaller space between the glomerular and the glomerular capsule of the kidney were observed in the PAX4^−/−^ rabbits; (4) no obvious histopathological changes were observed in the spleen of the PAX4^−/−^ rabbits; (5) the boundary between the visual cells layer and the bipolar cell layer was not clear in the retina of the PAX4^−/−^ rabbits, and cells in the ganglion cells layer showed characteristics of hyperplasia and vacuolation; (6) inflammatory cells infiltration, collagen fibers hyperplasia, adipocytes ectopic deposition, and uneven muscle fiber thickness were shown in the skeletal muscle of the PAX4^−/−^ rabbits; (7) similar characteristics were also observed in the cardiac muscle of the PAX4^−/−^ rabbits, including cardiac muscle cell steatosis and thickened myocardial fibers. Significantly, the PAX4^+/−^ rabbits only exhibited slight hepatocyte steatosis and vacuolation of renal tubular epithelial cells. These results demonstrated that DM associated phenotypes were exhibited in the PAX4^−/−^ rabbits by inactivation of *PAX4* gene.

## Discussion

In this study, we successfully generated a *PAX4* gene KO rabbit model through CRISPR/Cas9 system, and large fragment deletions of the *PAX4* gene between two sgRNA targeting sites were detected in the *PAX4* KO rabbits. To our knowledge, *PAX4* gene is a crucial transcription factor for β cell development and insulin secretion (Sosa-Pineda *et al.* 1997; Sosa-Pineda 2004). This was confirmed in these *PAX4* KO rabbits, due to complete damage of *PAX4* gene structure and function caused by large fragment deletions, destroyed, incomplete pancreatic islet structure, decreased numbers of insulin-producing β cells and increased numbers of glucagon-producing α cells were observed in the KO rabbits, leading to persistent hyperglycemia and lethality of the PAX4^−/−^ rabbits. Moreover, previous studies have shown that pancreatic β cells dysfunction causes diabetes and persistent hyperglycemia, leading to long-term health complications (Brun and Gauthier 2008; Leslie *et al.* 2016). This was further proved in our *PAX4* KO rabbits, for typical DM associated phenotypes were also observed in the PAX4^−/−^ rabbits, including diabetic nephropathy, hepatopathy, myopathy and cardiomyopathy. Taken together, these results showed that mutation of *PAX4* gene is sufficient to disrupt gene function of *PAX4* gene in rabbit.

As far as we know, this is the first report of a *PAX4* gene KO model in rabbit. Compared with the WT littermates, the PAX4^−/−^ rabbits showed severe hyperglycemia and high death rates (100%), and all the PAX4^−/−^ rabbits died within 4 days after birth. All these phenomenons observed in the PAX4^−/−^ rabbits were consistent with the previous studies carried out in the *PAX4* gene KO mice (Sosa-Pineda *et al.* 1997). Additionally, previous studies also showed that PAX4^+/−^ mice exhibit insulin-producing β cells and do not develop diabetes, indicating that a single copy of *PAX4* is sufficient to promote normal β cells development and function (Sosa-Pineda *et al.* 1997; Sosa-Pineda 2004). Similar results have been concluded in this study, the PAX4^+/−^ rabbits did not exhibit any obvious abnormalities, with normal body weight and blood glucose, and survived to adulthood. However, the results of H&E staining and immunohistochemistry of pancreas sections showed that the PAX4^+/−^ rabbits also exhibited destroyed, incomplete pancreatic islet structure, decreased numbers of insulin-producing β cells and increased numbers of glucagon-producing α cells. But these changes in the PAX4^+/−^ rabbits were not as serious as in the PAX4^−/−^ rabbits, with the trend of slighter changes in the PAX4^+/−^ rabbits with smaller fragment deletions of the *PAX4* gene. However, based on the clinical characteristics of human MODY9, we speculate that it may take some time for the PAX4^+/−^ rabbits to develop DM associated phenotypes. Therefore, our next plan is to continue to feed these PAX4^+/−^ rabbits, to observe whether the heterozygotes could develop hyperglycemia and other DM associated phenotypes, and to investigate the possibility of this rabbit model used for DM gene therapy. At the same time, we will mate the heterozygotes with normal rabbits to observe their fertility. Furthermore, mutations of *PAX4* gene seem to increase DM risk, thus this model may also be used to investigate the influence of other factors on the onset of DM.

In conclusion, this is the first report of a *PAX4* gene KO model in rabbits, including characteristics of growth retardation, persistent hyperglycemia, decreased number of insulin-producing β cells, increased number of glucagon-producing α cells and typical DM associated phenotypes. This novel *PAX4* KO rabbit model will be useful for validating important pathological mechanisms, identifying novel therapeutic targets and screening new drugs for human DM.
