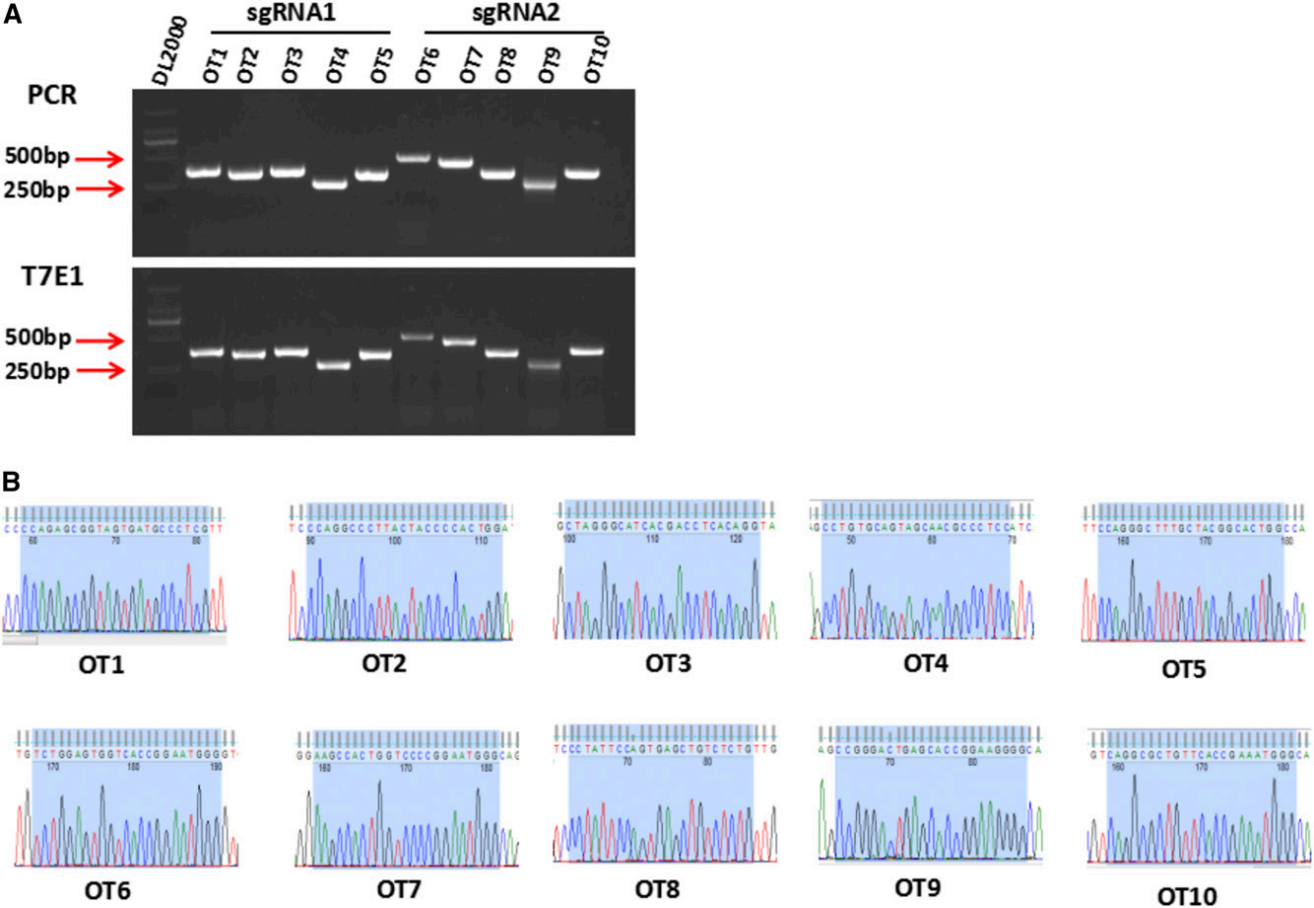# Corrigendum

**DOI:** 10.1534/g3.119.400079

**Published:** 2019-03-28

In the article by Y. Xu, Y. Wang, Y. Song, J. Deng, M. Chen, H. Ouyang, L. Lai, and Z. Li (*G3: Genes|Genomes|Genetics* 8(8): 2833-2840) entitled “Generation and Phenotype Identification of *PAX4* Gene Knockout Rabbit by CRISPR/Cas9 System”, there was an error in Figure 2A, which erroneously labeled two DNA fragments of D2000 DNA ladder as 500bp and 200bp. Figure 2 has been corrected to reference 500bp and 250bp.

**Figure 1:**